# A Concise Review of the Recent Structural Explorations of Chromones as MAO-B Inhibitors: Update from 2017 to 2023

**DOI:** 10.3390/ph16091310

**Published:** 2023-09-15

**Authors:** Reshma Susan Ipe, Sunil Kumar, Feba Benny, Jayalakshmi Jayan, Amritha Manoharan, Sachitra Thazhathuveedu Sudevan, Ginson George, Prashant Gahtori, Hoon Kim, Bijo Mathew

**Affiliations:** 1 Department of Pharmaceutical Chemistry, Amrita School of Pharmacy, Amrita Vishwa Vidyapeetham, AIMS Health Sciences Campus, Kochi 682041, India; ipereshma@gmail.com (R.S.I.); solankimedchem@gmail.com (S.K.); febacbenny@gmail.com (F.B.); jayalakshmi5599@gmail.com (J.J.); manoharanamritha99@gmail.com (A.M.); sachithrasudevan22@gmail.com (S.T.S.); ginsongeorge239@gmail.com (G.G.); 2 School of Pharmacy, Graphic Era Hill University, Dehradun 248007, India; pgahtori@gehu.ac.in; 3 Department of Pharmacy, and Research Institute of Life Pharmaceutical Sciences, Sunchon National University, Suncheon 57922, Republic of Korea

**Keywords:** chromones, neurodegenerative disorders, monoamine oxidase-B, structure-activity relationship, Parkinson’s disease, Alzheimer’s disease

## Abstract

Monoamine oxidases (MAOs) are a family of flavin adenine dinucleotide-dependent enzymes that catalyze the oxidative deamination of a wide range of endogenous and exogenous amines. Multiple neurological conditions, including Parkinson’s disease (PD) and Alzheimer’s disease (AD), are closely correlated with altered biogenic amine concentrations in the brain caused by MAO. Toxic byproducts of this oxidative breakdown, including hydrogen peroxide, reactive oxygen species, and ammonia, can cause oxidative damage and mitochondrial dysfunction in brain cells. Certain MAO-B blockers have been recognized as effective treatment options for managing neurological conditions, including AD and PD. There is still a pressing need to find potent therapeutic molecules to fight these disorders. However, the focus of neurodegeneration studies has recently increased, and certain compounds are now in clinical trials. Chromones are promising structures for developing therapeutic compounds, especially in neuronal degeneration. This review focuses on the MAO-B inhibitory potential of several synthesized chromones and their structural activity relationships. Concerning the discovery of a novel class of effective chromone-based selective MAO-B-inhibiting agents, this review offers readers a better understanding of the most recent additions to the literature.

## 1. Introduction

According to epidemiological data, Parkinson’s disease (PD) and Alzheimer’s disease (AD) are the most prevalent neurological illnesses. These conditions substantially negatively impact the suffering of individuals, relatives, caretakers, and the community. Unfortunately, only palliative treatments are currently available, which makes the design and creation of novel medications necessary [1,2,3]. AD is a neurological condition primarily affecting older people and is characterized by memory loss and dementia [4,5]. A range of illnesses, mostly related to neuronal cells in the human brain, are called neurodegenerative diseases. These disorders, often age-dependent, can be broadly characterized by the gradual degradation of the framework and functioning of the central or peripheral nervous systems [6,7,8,9]. Because neuronal cells are the basic units of the neurological system, they seldom reproduce or replenish themselves, and neuronal destruction or demise results in an inevitable loss of memory and cognitive impairments in people; however, under rare circumstances, it could lead to impairments in movement, speech, and breathing [10].

The World Health Organization (WHO) has projected that 50 million individuals will live with dementia worldwide by 2020, with approximately 10 million new cases occurring yearly. This number is expected to increase to 152 million by 2050. According to the WHO, AD may be a factor in 60–70% of dementia cases [6,11]. Despite numerous investigations into novel treatments in various phases of clinical trials, no effective therapies cure or reduce the progression of neurodegenerative illnesses [12,13,14]. Existing therapies may help alleviate most associated psychological and physical symptoms [12,15].

Monoamine oxidase (MAO) is a flavin adenine dinucleotide-dependent enzyme that is mainly found on the outer surface of mitochondria and is responsible for the oxidative breakdown of monoamines, including neurotransmitters such as dopamine, norepinephrine, as well as 5-hydroxytryptamine (serotonin) [16,17,18,19]. MAO-A and MAO-B are two subtypes that differ in tissue distribution, substrate particularity, susceptibility to particular inhibiting agents, and amino acid sequence [20,21]. Specifically, MAO-A deaminates noradrenaline, whereas MAO-B preferentially deaminates phenylethylamine, serotonin (5-hydroxytryptamine), and benzylamine [22]. MAO-B is primarily present in glial cells in the brain [23], whereas MAO-A is found in noradrenergic, serotonergic, and dopaminergic nerves and extra-neuronal compartment terminals [24]. Specific MAO-B blockers have been employed with levodopa to treat PD, whereas specific MAO-A-inhibiting agents have been utilized as antidepressants and anxiolytics [25,26,27,28]. MAO catalyzes the generation of hydrogen peroxide (H_2_O_2_) and reactive oxygen species (ROS), which may lead to oxidative stress and cell damage, ultimately leading to the progression of neurodegenerative disorders (ND); therefore, the concurrent inhibition of MAO may provide additional advantages for the treatment of ND [29,30,31]. MAO inhibitors are uncommon in clinical settings because only two medications, rasagiline and selegiline, have been approved for use as MAO-B blockers [32]. Both inhibitors have irreversible effects and are used to treat PD. Another MAO-B inhibitory compound, safinamide, has been clinically tested and functions as a reversible blocker [33]. Various scientific communities have focused on searching for novel MAO-B blockers with characteristics similar to those approved because of the limited number of -B blockers accessible for clinical use [34].

**Figure 1:**
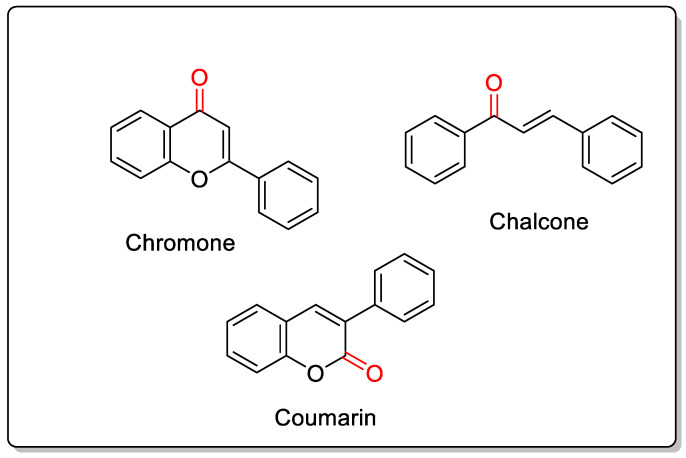
Structurally similar oxygen-containing MAO-B inhibitors.

This review is solely concerned with substances belonging to the chromone class with MAO-inhibitory capabilities. The well-known MAO-B blockers chalcones and coumarins share structural similarities with chromones (Figure 1).

As shown in Figure 1, the structures of chalcones and coumarins are closely related to those of chromones. The MAO-B inhibitory properties of coumarins and chalcones have been reported in numerous studies [35,36,37,38,39]. Considering the similarities between these structures and chromones, further studies are needed to identify novel therapeutic candidates.

## 2. Chemistry of Chromones

**Figure 2:**
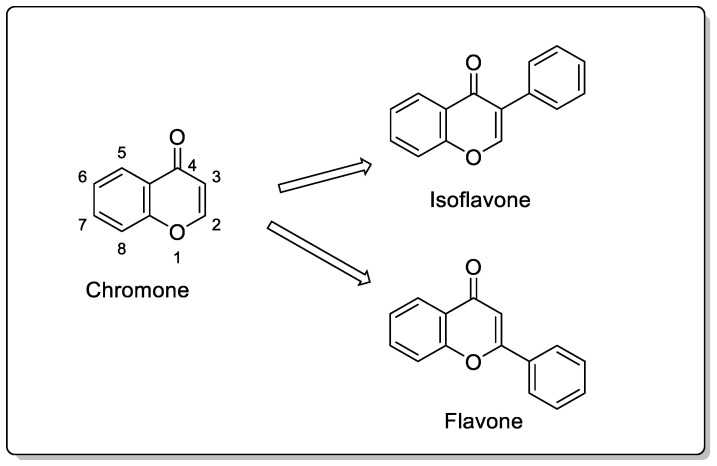
Structure of a chromone, isoflavone, and flavone.

Chromones (4H-chromen-4-one,4H-1-benzopyran-4-one) are a significant class of oxygen-containing heterocyclic compounds with a benzoannelated-pyrone ring [40,41]. They belong to the flavonoid family, which includes isoflavones and flavones (Figure 2).

**Scheme 1:**
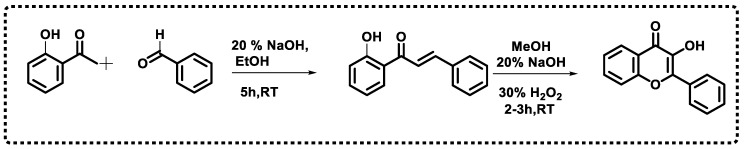
The common route involved in the preparation of chromone scaffold.

The development of substituted chromone analogs has an extensive background and is of great interest [42]. One of the most frequently used methods for producing chromones is the Claisen–Schmidt condensation of an aromatic aldehyde with an *ortho*-hydroxyarylketone, followed by cyclization [43,44] (Scheme 1). The small polar surface area (PSA) of chromones promotes blood–brain barrier crossing, which is primarily responsible for chromone-derived substances’ ability to exert their effects on the CNS. Numerous heterocyclic scaffolds have been considered for developing novel MAO-B inhibitors because isoform selectivity is a major concern. Isomers of coumarin, called chromones (4H-1-benzopyran-4-one), constitute the flavone backbone. The chromone ring system is regarded as a favored scaffold owing to its range of pharmacological and biological effects and the minimal risk of toxicity associated with chromone derivatives. Compounds derived from chromone scaffolds inhibit MAO. The AChE inhibitory potential of chromones was also investigated [45,46]. As dopamine D_2_ receptor agonists, chromones have been shown to mimic the actions of dopamine in the body, making them a potentially useful class of drugs for treating PD [47]. Chromones have been shown to have a significant capacity to adhere to adenosine receptors and have been studied clinically for managing PD and various CNS disorders [48,49,50,51,52].

Studies investigating the specificity of chromones have discovered that the methyl substitution on the chromone ring seemed more crucial for MAO-B inhibition than that of MAO-A. The current review, therefore, focuses on investigating the structure–activity relationship (SAR) and different methods of synthesis of various substituted chromones to determine how they affect MAO inhibitory activity.

## 3. SAR Studies of Chromone as MAO-B Inhibitors

**Scheme 2:**
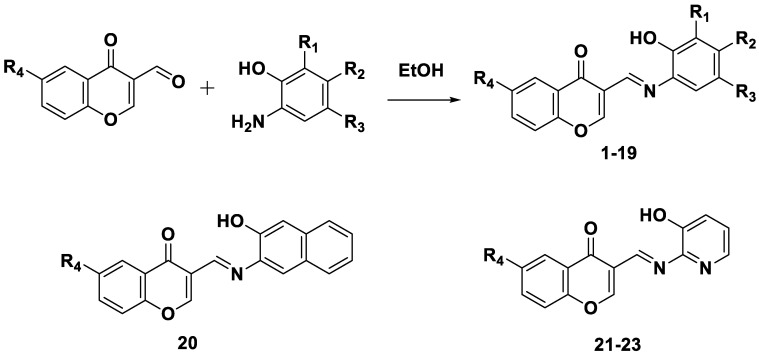
Synthesis of chromones based on Schiff’s base compounds (**1**–**23**).

Li et al. (2017) reported the discovery of multipurpose chromone-containing ligands with the potential for MAO inhibition, reaction with β-amyloid, chelation with metals, antioxidant activity, and regulation of reactive oxygen species [29]. They employed phenolic hydroxyl groups and Schiff bases as metal chelators. Target multifunctional ligands (MLs) were modified with phenolic substituents because they are known to have antioxidant properties. Several unique chromone derivatives were obtained and positioned at the C_3_ position of the pyrone ring as a result of the functionalization of the chromone nucleus. The compounds were prepared by condensing an aldehyde with an aromatic amine in ethanol under reflux conditions, which is the traditional technique for imine synthesis shown in Scheme 2.

**Figure 3:**
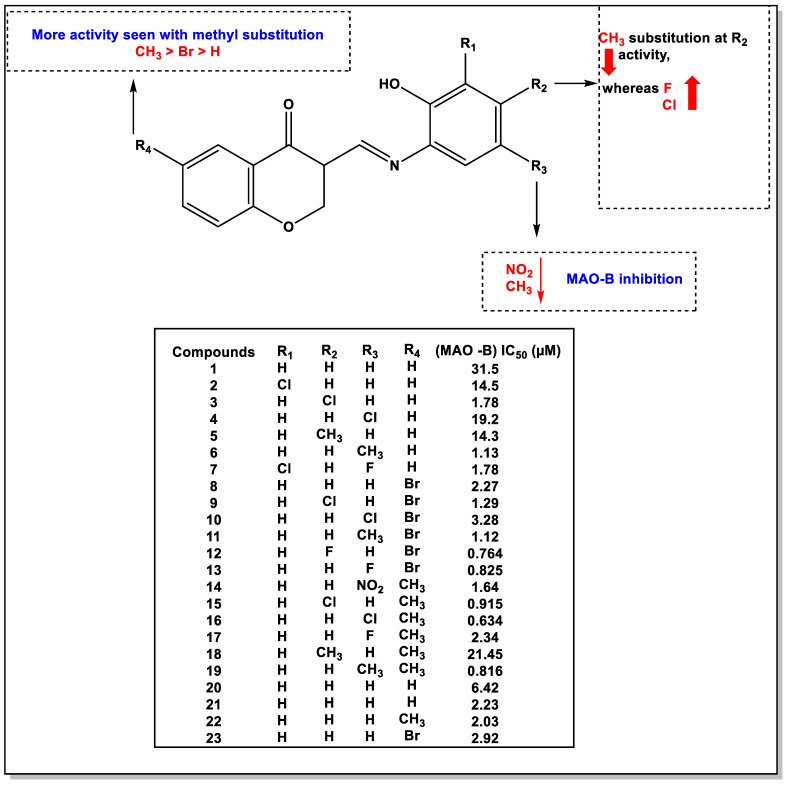
SAR study of chromone derivatives as MAO-B inhibitors.

Figure 3 shows that adding different substituents to the phenyl ring or chromone moiety improved hMAO-B activity compared to compound **1** with no substitution (IC_50_ = 31.5 µM). It was found to be more effective in inhibiting MAOs when the bromine substitution was at the R_4_ position of the chromone, as in the cases of **8**–**13** and **12,** with the F substitution at the R_2_ position of the phenyl ring. Introducing the methyl group in the R_4_ position of compounds **14**–**19** showed better activity than other analogs. Still, compound **18** showed the least inhibitory activity, with an IC_50_ value of 21.45 µM for hMAO-B, with the CH3 group at the R_2_ position. The highest inhibitory activity was demonstrated by compound **16**, which had a methyl substitution at the R_4_ position, and in the R_3_ position of the phenyl ring, a chlorine group was introduced. The IC_50_ of 0.634 µM is roughly 12 times more active than the standard iproniazid (IC_50_ = 7.98 µM). A complete loss of inhibitory activity was observed when Cl group **15** was replaced with NO_2_ group **14** at the R_2_ position of the phenyl ring. Compounds **12, 13, 15, 16,** and **19** demonstrated potent MAO inhibitory activities. The phenyl ring in the proposed compounds was changed to a different naphthalene ring **20** and pyridine rings **21**–**23**, which were found to have less potent activity. According to this study, it can be concluded that the NO_2_ and CH_3_ groups at R_3_ decrease MAO activity, whereas halogens such as chlorine or fluorine can cause a marked increase in activity. More potency was found with a methyl substitution at R_4_.

**Figure 4:**
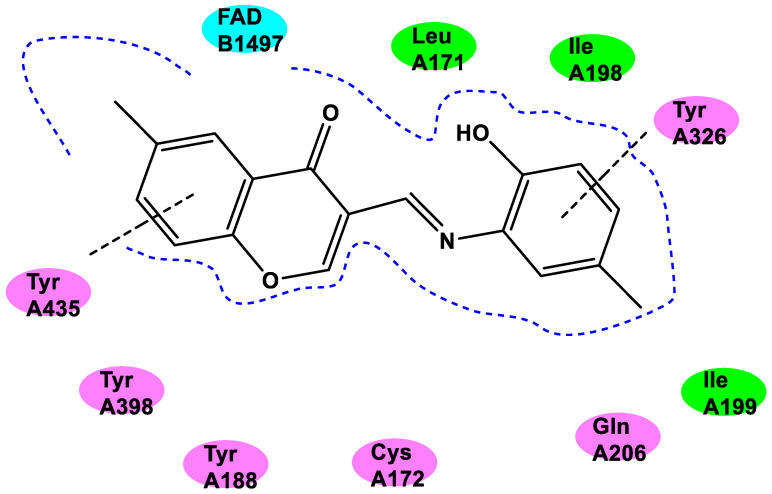
Docking study of compound **19.** Green, hydrophobic amino acid residue; purple, hydrophilic amino acid residue; blue, FAD.

A docking study was performed using this protein (PDB ID:2V61). Figure 4 explains the two-dimensional interaction, which showed that the FAD cofactor was situated near the chromone moiety of **19** and that the phenyl group of **19** had a π-π stacking interaction with Tyr435 at a distance of 3.18 Å. Tyr326 was involved in a π-π stacking contact with the amide carbonyl of **19** separated by a distance of 3.49 Å. Additionally, the hydrophobic pocket in the entrance cavity created by Leu171, Ile198, and Ile199 was occupied by the 2-amino-4-methylphenol moiety in compound **19**. These interactions may account for compound **19**’s effective inhibitory activity against MAO-B.

**Figure 5:**
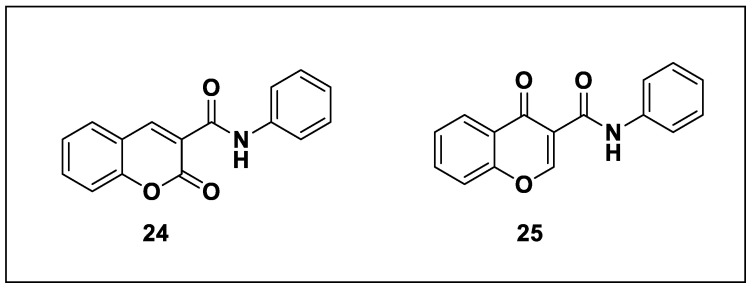
Coumarin-3-phenylcarboxamide (**24**) and chromone-3-phenylcarboxamide (**25**).

Fonseca et al. (2017) developed a set of novel coumarin and chromone derivatives and tested their biological activities against MAO-B [1]. With a focus on the lead optimization and under the guidance of the data obtained thus far, they carried out a detailed SAR examination of the two structural isomers. Compounds **34** and **54** were the most effective, selective, and reversible non-competitive MAO-B inhibitors. Searching for novel chemical compounds with pharmacological activities primarily involves using heterocyclic compounds. Benzopyrones are primarily coumarins and chromones. Chromones and coumarins are abundant and have useful therapeutic activities, such as antioxidant, anti-inflammatory, cardioprotective, and antibacterial effects. Coumarin-3-phenylcarboxamide **24** and chromone-3-phenylcarboxamide **25** (Figure 5) are desirable structures for the rational creation and identification of novel MAO-B inhibitors, according to Fonseca et al. and his research team.

**Scheme 3:**
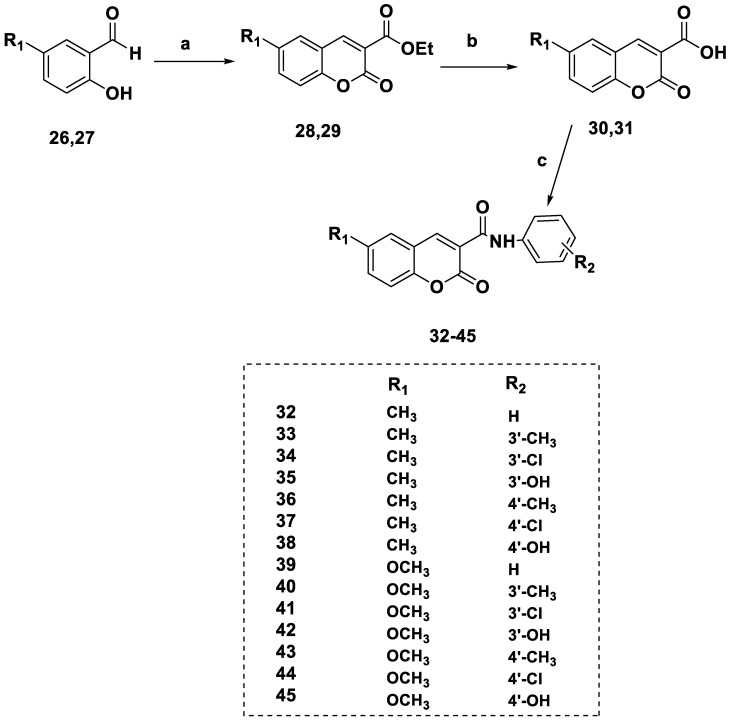
Synthesis of the coumarin derivatives **32**–**45**. Reagents and conditions: (a) diethyl malonate, EtOH, piperidine, reflux, overnight; (b) NaOH (0.5% aq. ethanol), reflux, 4 h; (c) EDC, DMAP, DCM, substituted phenylamine, 0° to room temperature (rt), 4 h.

**Scheme 4:**
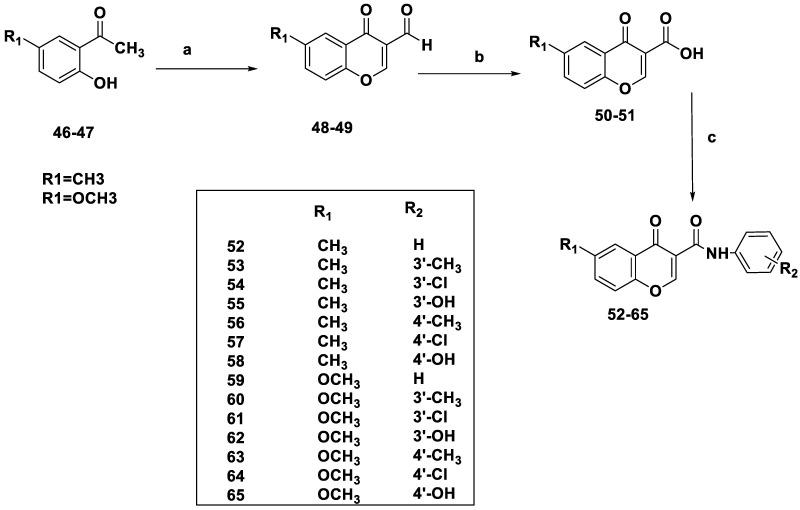
Synthesis of chromone-3-phenylcarboxamide **52**–**65**. Reagents and conditions: (a) POCl_3_, DMF, −10 °C, 15 h; (b) H_3_NSO_3_, NaClO_2_, 0 °C, 12 h; (c) POCl_3_, DMF, phenylamine, rt, 1–5 h.

A library of novel coumarins **32**–**45** (Scheme 3) and chromones **52**–**65** (Scheme 4) was synthesized and evaluated for MAO-B inhibition. Scheme 3 shows the synthesis of coumarin derivatives by treating salicylaldehyde with diethylmalonate to obtain coumarin-3-carboxylates **28** and **29** and coumarin carboxylic acids **30** and **31**. The coumarin-3-carboxamide derivatives **32**–**45** were created via the EDC-induced coupling of compounds **30** and **31** with phenylamines.

Scheme 4 explains the synthesis of chromone derivatives by treating acetophenone with phosphoryl chloride (POCl_3_) and N,N-Dimethyl formamide (DMF) at −10 °C for 15 h to obtain chromone-3-carbaldehydes (**48** or **49**) followed by oxidation of the formyl group with sodium chlorite to obtain chromone carboxylic acids (**50**–**51**). The synthesis of chromone-3-carboxamide derivatives (**52**–**65**) required the in situ formation of an acyl chloride intermediate, followed by the inclusion of a suitable phenylamine.

**Figure 6:**
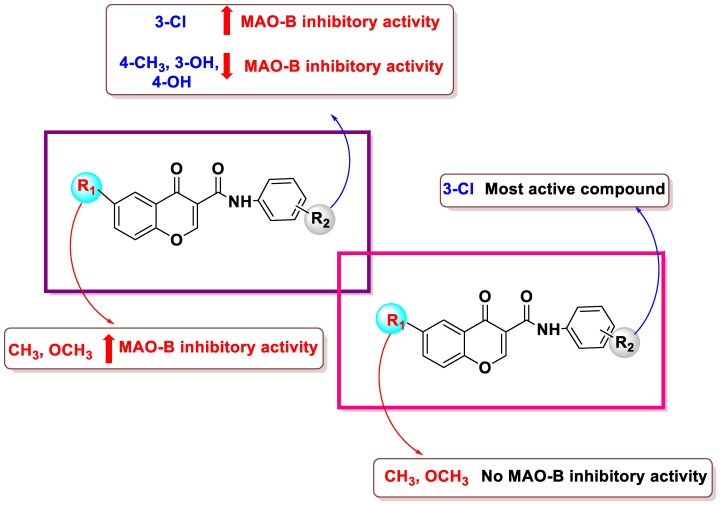
SAR study of chromone and coumarin derivatives as MAO-B inhibitors.

SAR analysis was conducted in response to earlier studies examining substituents’ effects at comparable positions on the isomeric scaffolds of coumarin- and chromone-based compounds as MAO inhibitors. Adding 6-CH_3_ or 6-OCH_3_ substituents to benzopyrone-3-phenylcarboxamide scaffolds led to strong MAO-B inhibition (Table 1). However, the 6-methylcoumarin derivatives (**32**–**38**) were more potent than their 6-methoxy analogs (**39**–**45**). The addition of a 6-methyl (**52**–**58**) or 6-methoxy (**39**–**45**) group to the chromone framework had no appreciable impact on the MAO-B inhibitory activity, except for compound **52** (which lacks substituents on the exocyclic ring). Derivatives with *meta*-alternatives on the exocyclic ring were shown to have increased potency in both series. Compounds **34**, **54,** and **61**, all of which had *m*-chlorine substituents, were the major active compounds in both sets, with IC_50_ values of 5.07, 4.2, and 3.94 μM, respectively. The addition of substituents in the *para*-group resulted in decreased activity, except for compound **43** (IC_50_ = 19.43 μM), which had a *p*-CH_3_ group, as compared to compound **40** (IC_50_ = 47.24 μM), which had a *m*-methyl group. The activity was reduced by the influence of an OH group at either the *meta-* or *para*-position (Figure 6). The location of the benzopyrone carbonyl group on the pyrone ring did not appear to have a significant effect, although MAO-B inhibition was a function of this group. All compounds under investigation were selective for MAO-B (Table 1).

The Reis collection of chromone was developed, synthesized, and tested for MAO and choline esterase inhibition by Reis et al. [53]. Briefly, the chromone moiety was modified by adding a phenylcarboxamide group at position C_2_ or C_3_ and an acrylate moiety with a tertiary amine function at position C_6_. The potential byproducts of acrylate side-chain hydrolysis were also determined, and their biological activities were assessed in vitro. Additionally, testing was conducted on the most potent compounds, which were tested to determine their ability to permeate the blood–brain barrier, enzyme inhibition, kinetics and mechanisms, drug-like properties, and cytotoxicity profiles. Using models based on the crystal shapes of the targets, molecular modeling studies were performed to understand the interactions between the compounds and targets. The protein (PDB ID:2V5Z) was used as the receptor model for hMAO-B.

**Scheme 5:**
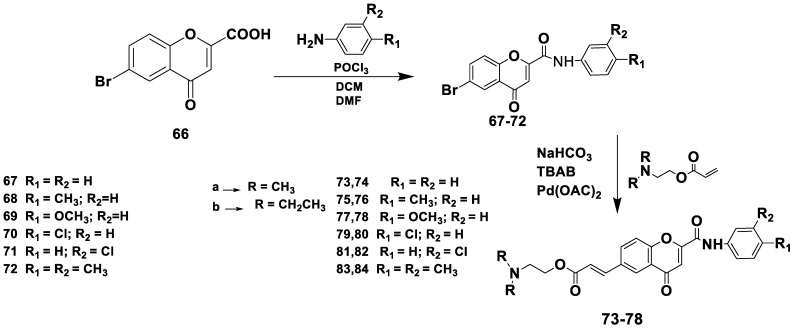
Synthesis of three substituted amide-based chromones.

Scheme 5 shows the synthesis of chromone-2-phenylcarboxamide derivatives by treating 6-bromo-4-oxo-4H-chromene-2-carboxylic acid with phenylamine derivatives, followed by microwave-assisted Pd(II)-catalyzed Heck cross-coupling using 2-(dimethylamino)ethyl acrylates **73**, **75**, **77**, **79**, **81**, and **83** or 2-(diethylamino)ethyl acrylates **74**, **76**, **78**, **80**, **82**, and **84**.

**Scheme 6:**
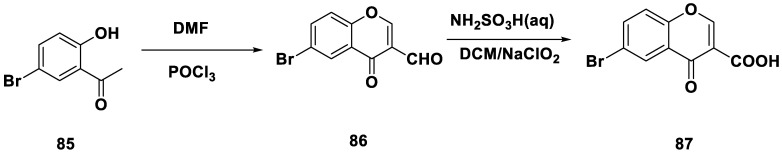
Synthesis of 6-bromo-chromone-3-carboxylic acid.

In Scheme 6, compound **86** (6-bromo-4-oxo-4H-chromene-3-carbaldehyde) was prepared from the starting material 5′-bromo-2′-hydroxyacetophenone **85** using POCl_3_-induced cyclization followed by subsequent oxidation in the presence of sodium chlorite and sulfamic acid to obtain bromo-4-oxo-4H-chromene-3-carboxylic acid (**87**). From **87,** the derivatives **88**–**93** were prepared by the condensation of phenylamine derivatives, followed by a microwave-assisted Pd(II)-catalyzed Heck cross-coupling reaction, as described in Scheme 5, to obtain chromone 3-phenylcarboxamides **94**–**105**.

**Scheme 7:**
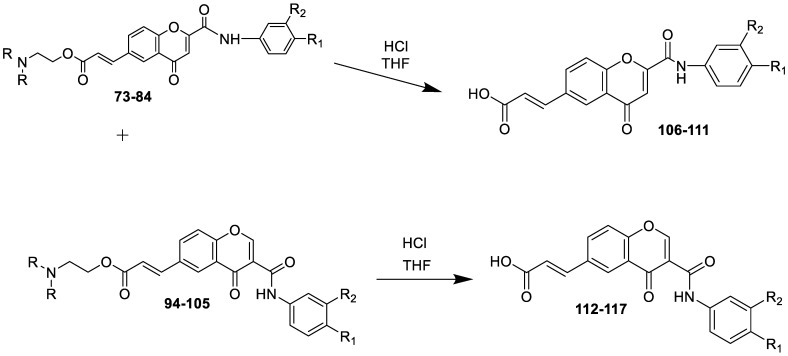
Synthesis of chromone 3-phenylcarboxamides derivatives.

Scheme 7 shows the synthesis of chromones **106**–**111** and **112**–**117** prepared by the acid hydrolysis of chromone 2-phenylcarboxamides **73**–**84** and chromone 3-phenylcarboxamides **94**–**105**.

**Figure 7:**
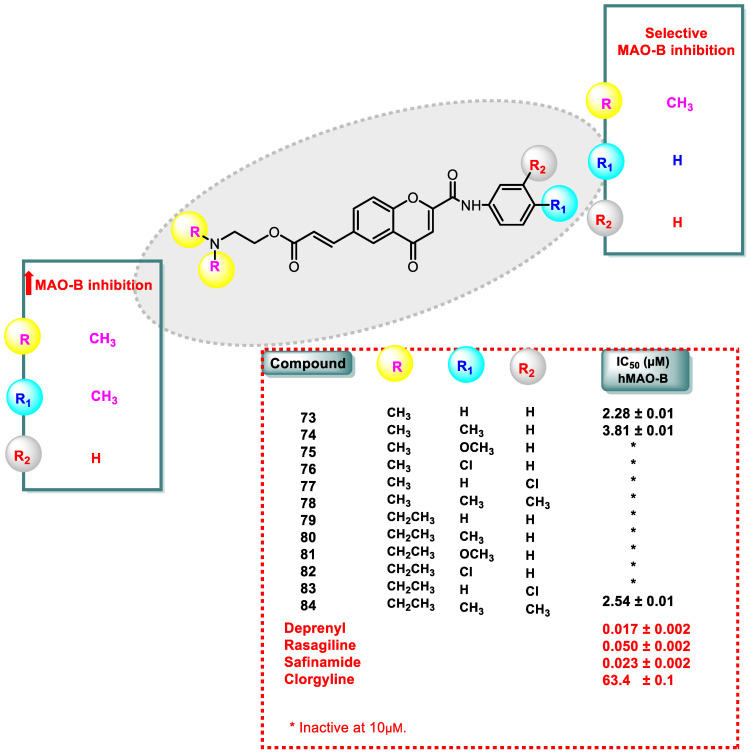
SAR study of chromone 2-phenylcarboxamide derivatives (**73**–**84**) and its MAO-B inhibitory activity.

Compound **73**, with two methyl (CH_3_) groups in the tertiary amine nucleus and no substituents on the exocyclic phenyl ring, specifically inhibited MAO-B with an IC_50_ value of 2.28 μM. Derivative **74** inhibited MAO-B, which has two -CH3 groups bound to the tertiary amine and a methyl group at the para position of the chromone exocyclic phenyl ring (Figure 7).

**Figure 8:**
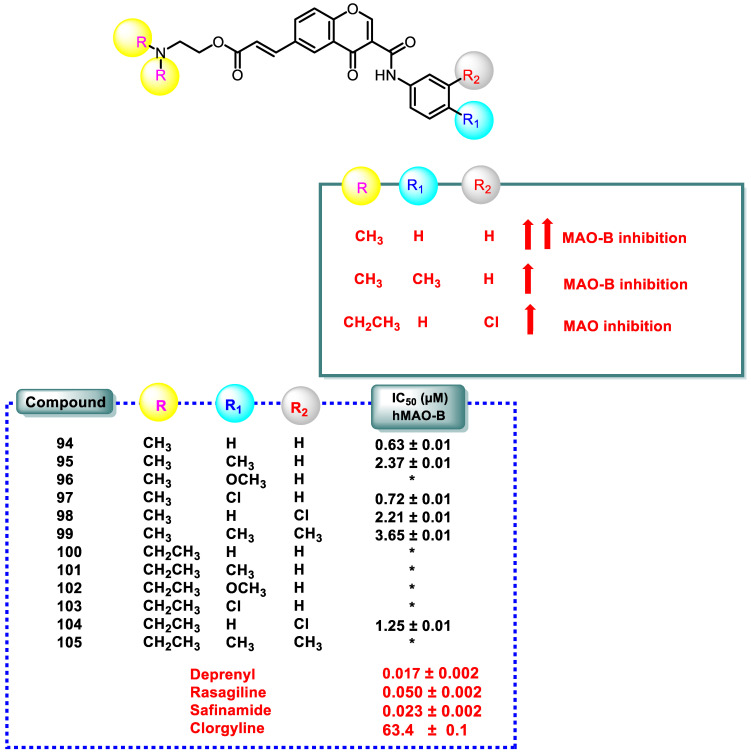
SAR study of chromone-3-phenylcarboxamide derivatives (**94**–**105**) and its MAO-B inhibitory activity. *, not determined.

Except for compounds **96**, **100**–**103**, and **105**, most of the chromone 3-carboxamide derivatives exhibited micromolar and sub-micromolar MAO-B-specific inhibition. In this sequence, the spatial volume of the substituent on the tertiary amine and/or in the exocyclic aromatic ring significantly influenced MAO-B inhibitory activity. Dimethyl-N-substituted compounds **100**–**105** were inert towards MAOs, except for compound **104**. Notable MAO-B inhibitory activity was observed for compound **94**, which had no derivatives on the exocyclic ring. The *p*-CH_3_ group on compound **95** allowed it to selectively inhibit MAO-B compared to MAO-A, with an SI value > 4.2 (Figure 8).

**Figure 9:**
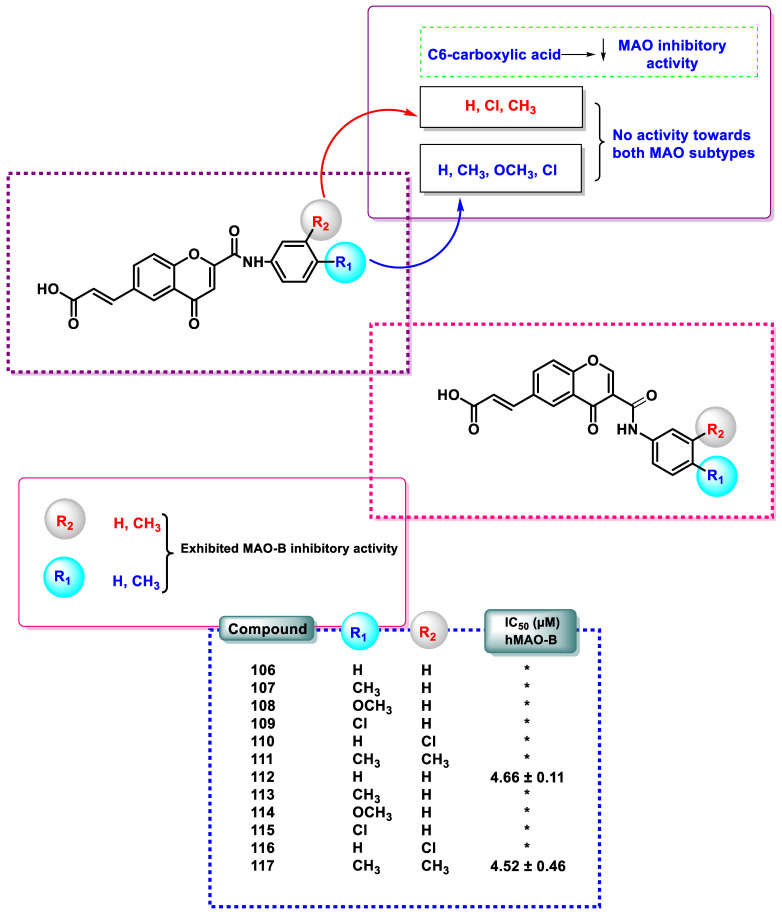
SAR study of chromone-2-phenyl and 3-phenyl carboxamide carboxylic acid derivatives **106**–**117** as MAO-B inhibitors. *, not determined.

The potential compounds **106**–**117**, synthesized by the hydrolysis of acrylate-substituted chromones, were examined for their MAO inhibitory potential (Figure 9). The results revealed that the presence of the C6-carboxylic acid group significantly reduced the inhibitory activity of MAO. In particular, the hydrolyzed compounds of chromone 2-phenylcarboxamide **106**–**111** showed no effect on either of the MAO isoforms. Compared to the related precursors, compounds **94** and **99**, chromone 3-phenylcarboxamide **112** and **117** exhibited micromolar MAO-B inhibitory activity.

Mpitimpiti et al. developed a novel series of 15 chromone derivatives and tested their MAO inhibitory activity in light of earlier investigations on the possible inhibition of MAO by chromone compounds [54]. This study strongly emphasized the third position vs. the potential of MAO inhibition concerning the effect of flexible side chain replacement.

**Scheme 8:**
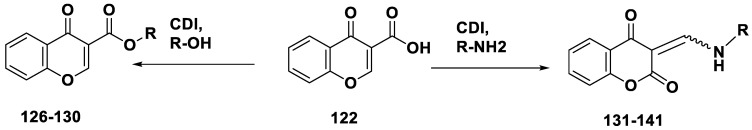
Synthesis of ester- and amino-based chromones.

Scheme 8 illustrates the preparation of ester **126**–**130** and amino **131**–**141** derivatives of chromone by treating the chromone 3-carboxylic acid **124** with aromatic/aliphatic amines and alcohols in the presence of carbonyldiimidazole (CDI). Although the target esters were successfully synthesized, it is not surprising that the reaction of **122** with the amine compounds produced chromane-2,4-diones (**131**–**141**). The results of the MAO inhibition studies revealed that the ester derivatives were ineffective MAO inhibitors; however, several chromane-2,4-diones showed promising MAO-B inhibition potencies. The most effective MAO-B inhibitor was compound **133,** with an IC_50_ value of 0.638 µM. Compound **131** is a reversible MAO-B inhibitor. However, compound **131** was a less potent MAO inhibitor than lazabemide, a reversible MAO-B-specific inhibitor (IC_50_ = 0.091 μM), assessed under comparable laboratory conditions. Similar to previously reported C6- and C7-substituted chromones, **135** had a much lower MAO-B-inhibiting potential. Chromones **118**–**120**, at least one order of magnitude more effective MAO-B inhibitors than **131**, serve as indicators.

For example, a group of chromone compounds with C6 and C7 substitutions were shown to be efficient reversible MAO-B inhibitors. These studies have led to the development of effective MAO-B inhibitors, including compounds **118**–**121**. Although some of these chromones also displayed IC_50_ values for MAO-A inhibition in the nanomolar range, these derivatives were specific inhibitors of the MAO-B isoform.

**Figure 10:**
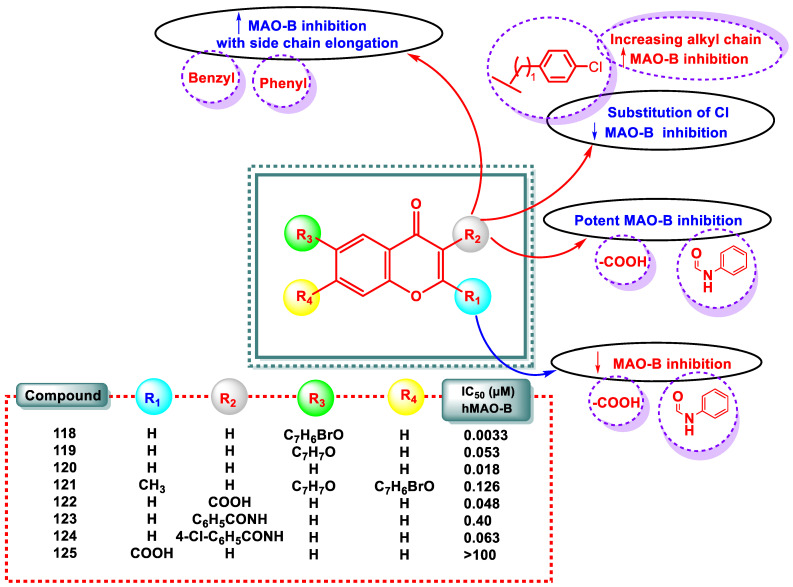
SAR study of chromone derivatives and the inhibition of MAO-B by chromone derivatives **118**–**125**.

Intriguingly, chromone substitution at position C5 resulted in modest MAO-B inhibition, in contrast to the C6- and C7-substituted derivatives [55]. Chromone 3-carboxylic acid **122** is an effective and selective MAO-B inhibitor (IC_50_ = 0.048 μM), despite that the COOH group being present in location 2 of the 4-pyrone nucleus resulted in a decrease in activity compound **125** [56,57,58]. Similarly, a phenylcarboxamide substitution at position 3 of the 4-pyrone nucleus resulted in significant MAO-B inhibition, with derivatives **123** and **124** exhibiting IC_50_ values of 0.40 and 0.063 μM (Figure 10) [57,58,59].

**Figure 11:**
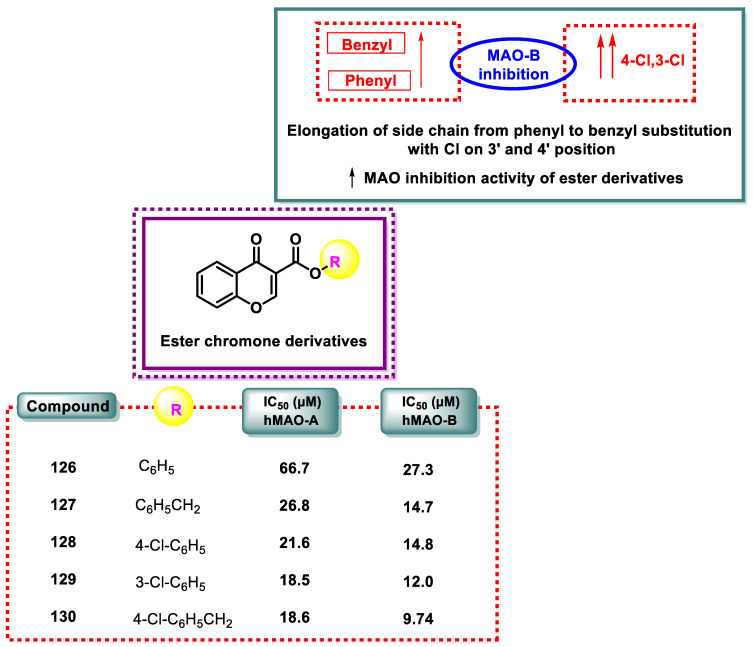
SAR study of ester chromone derivatives **126** and the inhibition of MAO-A and MAO-B.

Figure 11 illustrates the results of the MAO inhibition experiments. Ester derivatives often exhibit IC_50_ values between 18.6 and 66.7 μM and 9.74 and 27.3 μM, which shows that the ester analogs are poor MAO-A and -B inhibitors, as shown in Figure 11. The benzyl derivative was more effective than the phenyl analog when comparing the potencies of compound **127** (benzyl-substituted) and compound **126** (phenyl-substituted). The MAO inhibitory efficacy increased as the chain lengthened from phenyl to benzyl. A similar phenomenon was observed when the activity of derivative **128** was compared with that of compound **126**. The MAO inhibitory action was enhanced when comparing the 4-chlorophenyl substitution **128** to the phenyl side chain without substitution **126**. A comparison of compound **130** with compound **127** revealed a similar pattern. Compound **130** was a derivative of the 4-chlorobenzyl replacement. The introduction of chlorine had little to no impact on the MAO inhibitory action, as shown by comparing 4-chlorophenyl and 3-chlorophenyl substitutions **128** and **129**. Therefore, it can be deduced that extending the side chain from phenyl to benzyl and adding a Cl atom to position 3 or 4 of the side chain improved the MAO inhibitory activity of the ester analogs.

**Figure 12:**
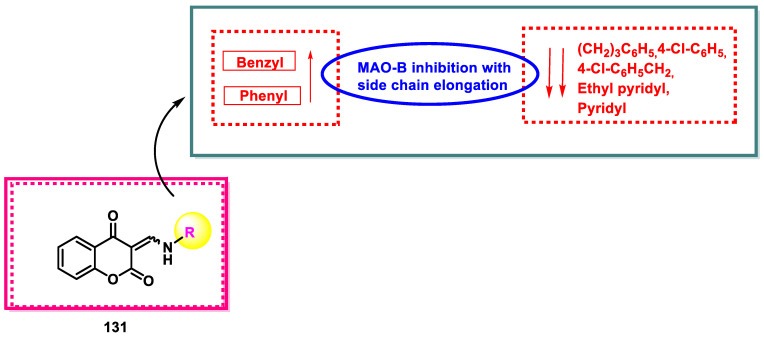
SAR study of 3-aminomethylidene-2,4-chromandiones.

Table 2 illustrates that chromane-2,4-diones are specific MAO-B inhibitors, with IC_50_ values of 0.638–16.66 µM. Compared to ester derivatives, chromane-2,4-diones often have higher MAO-B inhibitory effects. Compound **133**, with an IC_50_ value of 0.638 μM, is the most potent MAO-B inhibitor. Side chain elongation increased with MAO-B inhibition from compounds **132** to **133** (phenyl to benzyl); however, from compounds **134** to **135**, as chain elongation increased, MAO-B inhibitory activity decreased. Compounds **136, 137**, and **138** did not affect MAO-A or MAO-B, which could be attributed to adding a sterically large chlorine atom. When the MAO inhibitory activities of compounds **132**–**133** were compared with those of compounds **136**–**138**, it was concluded that Cl substitution decreased the MAO-B inhibitory activity. When comparing the compounds with benzyl/phenyl substitutions to those with pyridyl substitutions, the former showed superior inhibition (Figure 12). Chain elongation from pyridyl **140** to ethyl pyridyl **141** led to a two-fold increase in MAO inhibition compared to compounds with pyridine-containing side chains. The results also demonstrate that chromane-2,4-diones are more effective MAO-B inhibitors than ester chromone analogs, with an IC_50_ value of 0.638 µM for **133** compared to 14.7 µM for **127**. For instance, phenyl-substituted molecule **132** was approximately 28 times more potent than **126**. Similarly, the MAO-B inhibitory potencies of chromane-2,4-diones **133** and **137** were much greater than those of their corresponding ester derivatives **127** and **128**. The 3-aminomethylidene-2,4-chromandiones are inseparable mixtures of *E-* and *Z*-isomers, and the MAO inhibitory potencies indicated are those of the mixtures, which should be emphasized. According to a study by Cagide et al., the MAO inhibitory characteristics of four 3-(phenylamino)methylidene chromane-2,4-dione derivatives and chromane-2,4-diones are weak MAO-A inhibitors [59]. These compounds failed to suppress human MAO-A at the highest tested concentration of 10 μM. However, as reported by Cagide et al., the IC_50_ values for **132** (0.268 µM) and **137** (0.065 µM) to inhibit human MAO-B are considerably different from those found in the current study. Although the exact cause of this mismatch is unknown, it could be related to the different experimental methods employed to determine the MAO activity.

Over the past few decades, in both academic and professional contexts, molecular docking has been frequently used as a quick and affordable approach. There is still no easy and accurate way to quickly identify real ligands among a group of molecules or to precisely pinpoint the right ligand conformation inside the binding pocket of MAO, despite the fact that this discipline has had enough time to consolidate in many ways [60]. Based on the crystal structures of MAO-B in combination with reversible inhibitors, it is possible to predict that chromane-2,4-diones will bind to the active site of their moiety close to FAD, the most polar area. The chromane-2,4-dione moiety forms hydrogen bonds with the water molecules and amino acid residues. The 3-aminomethylidene side chain is intended to fill the entrance cavity, lined mostly with nonpolar residues. Interestingly, the binding positions of the trans- and cis-isomers are comparable. A π-π stacking interaction with Tyr-398 and a π-sulfur interaction with Cys-172 are both seen, despite normal hydrogen bonding (cis isomer). The side chain phenyl ring is stabilized by π-alkyl interactions with Ile-199 and Ile-316, while π alkyl interactions between the chromane-2,4-dione and Leu-171 and Ile198 and Cys-172 (trans-isomer) create the π alkyl interactions. By forming a carbon-hydrogen bond with the benzylic H of the side chain and the C_2_ carbonyl oxygen of chromane-2,4-dione, Ile-199 becomes even more significant. The phenolic oxygen of Tyr-326 additionally forms a carbon–hydrogen bond with benzylic H. Both isomers of **132** can bind to and interact with MAO-B; according to this analysis, the binding mechanisms and interactions are relatively comparable. Thus, both isomers are involved in MAO-B inhibition.

Takao et al. synthesized and assessed the MAO-A and MAO-B inhibitory properties of 18 2-styrylchromone derivatives [61]. In the prepared series, compound **150** had the greatest MAO-B inhibitory capacity and specificity with an –OCH_3_ substitution at R1 and a Cl substitution at R4. Compound **150** inhibited MAO-B competitively and reversibly. They used the pIC_50_ values of 2-styrylchromone derivatives to perform quantitative structure–activity relationship (QSAR) studies using the molecular operating environment (MOE) and dragon, which revealed significant relationships (*p* < 0.05). Through 3D-QSAR investigations using AutoGPA, based on a molecular field analysis method using MOE, 2-styrylchromone structures were investigated as valuable scaffolds. Based on these findings, the 2-styrylchromone moiety may be a good building block for novel MAO-B antagonists.

**Scheme 9:**
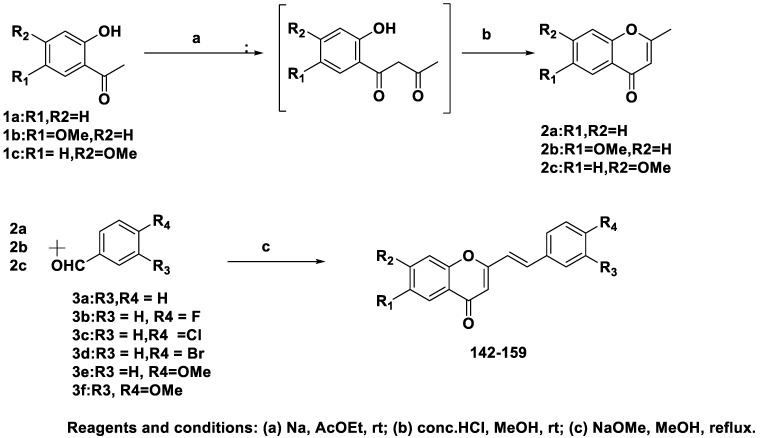
Synthesis of 2-styrylchromone derivatives.

Scheme 9 explains the synthesis of the 2-styrylchromone derivatives involved in the reaction of acetophenone derivatives with ethyl acetate, followed by intramolecular cyclization. The generated IIa-IIc then interacted with benzaldehyde derivatives IIIa–f in the presence of a base.

**Figure 13:**
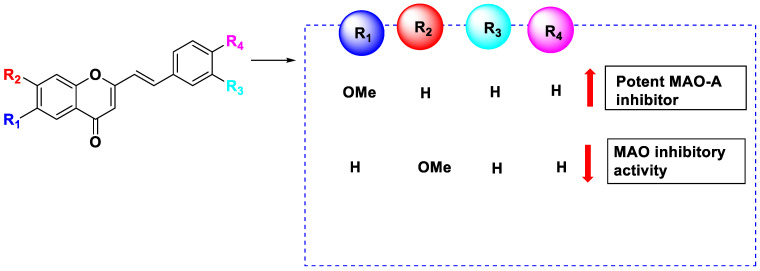
SAR study of 2-styrylchromone derivatives.

The inhibitory effects of 2-styrylchromone compounds **142**–**159** (Table 3) on MAO (A and B) were assessed. Modifications to R_3_ and R_4_ phenyl rings and R_1_ and R_2_ chromone rings showed several intriguing structure–activity relationships, as shown in Figure 13. We discovered that these compounds had inhibitory effects on MAO-A.

Compounds **142**–**146** and **148**–**151** displayed inhibitory activity, with compound **151** showing the most effective inhibition of MAO-A. Except for compound **144** vs. **150**, adding a methoxy substituent at R1 appeared to increase the MAO-A-inhibiting properties of compounds **142** vs. **147**, **143** vs. **149**, **145** vs. **151**, and **146** vs. **152**. All MAO-A inhibitory activities, including those of compounds **142** vs. **154**, **143** vs. **155**, **144** vs. **153**, **145** vs. **157**, **146** vs. **158**, and **147** vs. **159**, were reduced when the –OCH_3_ group was at position R_2_. The ability of the derivatives to inhibit MAO-B was assessed, and it was found that all derivatives inhibited MAO-B more potently than MAO-A. The most effective inhibitor was compound **150**, which showed inhibitory activity nearly 13 times greater than that of pargyline, used as a positive control. Compared to pargyline, compounds **144**–**146**, **149**, and **151** exhibited more potent inhibition, while compounds **142**, **143**, **148**, and **152**–**157** displayed comparable inhibition. Additionally, **146** vs. **152**, **143** vs. **149**, **144** vs. **150**, **145** vs. **151**, and **147** vs. **153** appeared to have stronger MAO-B inhibitory effects when the methoxy group at position R_1_ was substituted. Furthermore, compounds **156**, **157**, **158**, and **159** exhibited reduced MAO-B inhibitory effects when the methoxy group at position R_2_ was replaced.

Derivatives of 2-styrylchromone demonstrated strong and selective MAO-B blocking effects. The computational analysis of the statistical significance (*p* < 0.05) of each substituted group revealed that the methoxy substitution at position R_2_ substantially influenced both the selectivity and MAO-B blocking activity.

Wang et al. developed several donepezil-chromone hybrids **160**–**167** and **168**–**175** (Table 4) and tested their biological activity, including MAO inhibition and central nervous system penetration in vitro [62]. Molecular modeling studies were also conducted to evaluate the novel hybrid compounds’ interaction mechanism, structure–activity connections, and binding strategy.

**Scheme 10:**
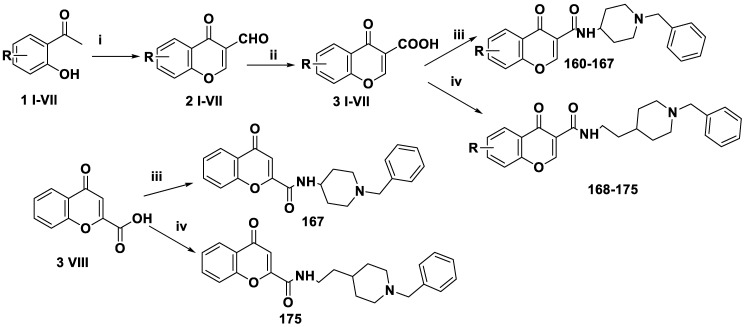
Synthesis of donepezil-chromone hybrids **160**–**167** and **168**–**175**. Reagent and condition, (i) POCl_3_, DMF, 0 °C, 2 h; (ii) NaClO_2_, NH_2_HSO_3_, CH_2_Cl_2_, 0 °C, 3 h; (iii) thionyl chloride, reflux; 1-benzylpiperidin-4-amine, K_2_CO_3_, CH_2_Cl_2_, rt, 8 h; (iv) thionyl chloride, reflux; 2-(1-benzylpiperidin-4-yl)ethanamine, K_2_CO_3_, CH_2_Cl_2_, rt, 8 h.

Scheme 10 shows the formation of donepezil-chromone hybrids **160**–**167** and **168**–**175**; they were prepared by treating 2-hydroxyacetophenone with phosphorus oxychloride (DMF) by a modified Harnisch procedure that resulted in the formation of 3-formylchromones, followed by oxidation with sulfamic acid and sodium chlorite to obtain the appropriate 4-oxo-4H-chromene-3-carboxylic acids, which were then treated with acetyl chloride and a catalytic amount of DMF in CH_2_Cl_2_ to produce acyl chloride and react with 1-benzylpiperidin-4-amine or 2-(1-benzylpiperidin-4-yl)ethanamine in CH_2_Cl_2_, resulting in the target compounds.

**Figure 14:**
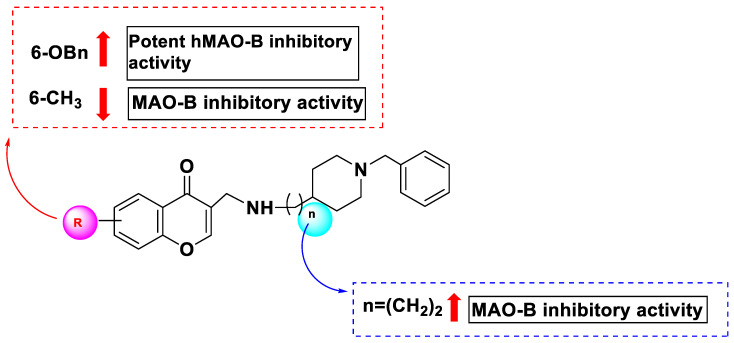
SAR of donepezil and chromone hybrids.

According to the SAR (Figure 14), hybrids containing a benzyloxy substitution at the sixth position of the chromone were more prone to have an inhibitory effect on hMAO-B. In particular, compound **162**, which had a benzyloxy substitution at C6 of the chromone, demonstrated the greatest inhibition with an IC_50_ value of 0.035 μM, demonstrating an activity that was nearly three and 200 times more than that of the standards pargyline and iproniazid, respectively. All other analogs produced less activity when other groups were added instead of the benzyloxy substitution. Compound **171**, which contained a methyl substitution at the same position, exhibited the weakest performance. Additionally, there was a strong correlation between the alkylene chain length and MAO-B inhibitory efficacy. Compound **170**, an excellent MAO-B inhibitor, had an N-ethylcarboxamide connection between the chromone and benzylpiperidine. This compound had an IC_50_ value of 0.272 μM and was 7.8 times less effective than its counterpart, compound **162,** with a carboxamide linker. These findings suggest that steric parameters affect the inhibitory effects of hMAO-B. Compound **170**, which showed the most balanced ability to selectively block MAO-B based on the findings of MAO inhibitory activity, is thought to be an appealing multi-purpose blocker for future research.

They assessed the ability of these hybrids to penetrate the BBB because brain crossing is a requirement for efficient anti-AD drugs. Di et al. developed an artificial membrane permeability test for the BBB (PAMPA-BBB) to achieve this. The three most efficient compounds (**162**, **169**, and **170**) were chosen as candidates to investigate the possible toxicological effects on rat pheochromocytoma (PC12) cells and the potential for therapy with these derivatives. Compound **170**, which had high MAO-B selectivity, was the most intriguing variant of the generated compounds. Compound **170** crossed the BBB and demonstrated minimal cell toxicity when tested in vitro on rat pheochromocytoma (PC12) cells. Overall, the multifunctional ligand **170**, which possesses balanced MAO-B inhibitory activities, may be considered a potential anti-AD target for future studies.

In the substrate cavity of the enzyme, the chromone moiety of compound **170** is located adjacent to the FAD cofactor. Its benzyloxy substitution interacts with Tyr435 and Tyr398 via π–π stacking interactions. Tyr326 interacted with the amide carbonyl of compound 172 via a hydrogen bond. In contrast, the benzylpiperidine moiety of compound 172 resides in the hydrophobic pocket formed by Pro102, Leu88, Ser200, Gly101, Glu84, Thr201, Ile199, and Ile316 in the entrance cavity. Additionally, Glu84 and the quaternary nitrogen present in piperidine form hydrogen bonds.

Takao et al. (2020) synthesized a collection of 2- and 3-(N-cyclicamino) chromone compounds and investigated their inhibitory activities against MAO [63]. Among the derivatives tested, including safinamide, which was employed as the control substance, compound **187iii**, 7-methoxy-3-(4-phenyl-1-piperazinyl)-4H-1-benzopyran-4-one, demonstrated a major antagonistic action, demonstrated the largest specificity towards MAO-B, and functioned reversibly and competitively. These findings implied that the lead substances for MAO-B inhibitory studies could be 3-(N-cyclicamino) chromone derivatives.

**Scheme 11:**
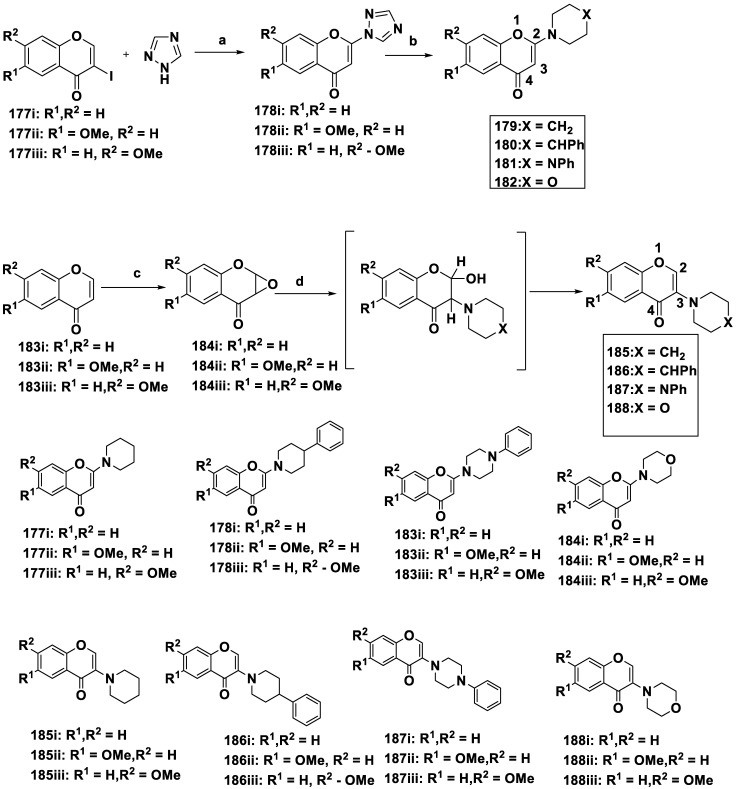
Synthesis of 2- or 3-(N-cyclic amino) chromone derivatives: (a) K_2_CO_3_, DMF, and at 80 °C; (b) cyclic amine, DMF, and at 80 °C; (c) H_2_O_2_, PhCH_2_N^+^(CH_3_)_3_OH^-^, Et_2_O, and at 0 °C; and (d) cyclic amine, CH_3_CN, rt.

The formation of 2- and 3-(N-cyclic amino) chromone analogs is shown in Scheme 11. When 3-iodochromone derivatives **177i**–**iii** were treated along with 1,2,4-triazole in the presence of K_2_CO_3_, DMF at 80 °C, it resulted in the generation of 2-(1,2,4-triazolyl)-chromone variants **178i**–**iii**, which then underwent a substitution reaction by interacting with the corresponding cyclic amine (DMF at 80 °C), forming the compounds of interest. Next, 3-(N-cyclicamino) chromone variants **185**–**188** were generated by the epoxidation of chromone derivatives **183i**–**iii** using an aqueous H_2_O_2_ solution under basic conditions to obtain 2,3-epoxychromone derivatives **184i**–**iii,** which reacted with the corresponding cyclic amine to form the target compounds (Table 5).

**Figure 15:**
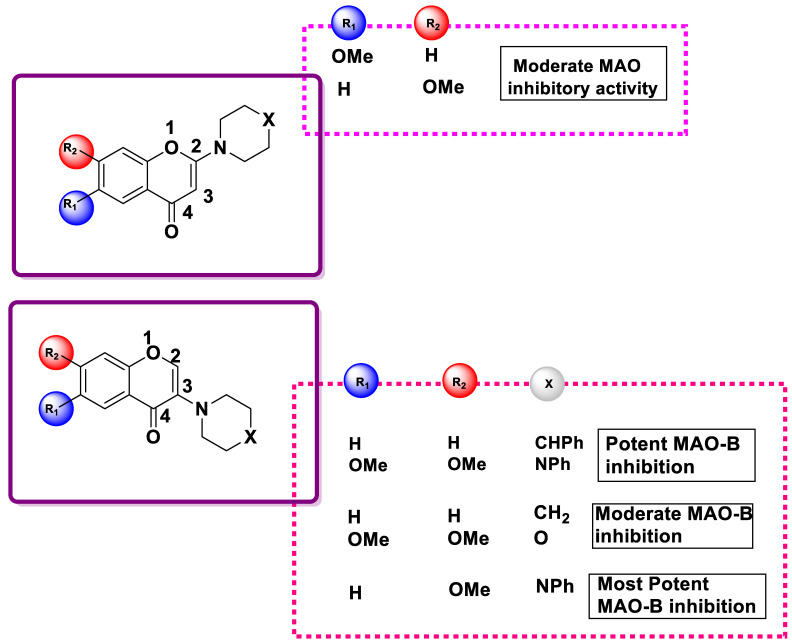
SAR of 2- and 3-(N-cyclic amino chromone derivatives).

Compounds **178ii, 179iii**, and **183ii** displayed equivalent IC_50_ concentrations for MAO-B and mildly inhibited MAO-A and MAO-B. Due to the smaller site action of MAO-A compared to MAO-B, compounds **186** and **187** did not show any inhibiting effects against MAO-A at a concentration of 100 μM. The derivatives **186i**, **186iii**, **187i**, and **187iii** effectively and selectively inhibited MAO-B. Compounds **185i**, **188ii**, and **188iii** moderately inhibited MAO-B. The most effective inhibition and MAO-B specificity were observed for compound **187iii**. Safinamide used as a positive control, and it was nearly three times more effective than compound **188iii** when its efficacy and selectivity were examined. Except for compounds **186ii** and **187ii**, the 3-(N-cyclic amino) chromone derivatives specifically inhibited MAO-B, whereas the 2-(N-cyclic amino) chromone derivatives did not. The current findings clearly distinguish the two sets of compounds, which is in line with earlier studies on chromone carboxylic acids and related amides, as well as the latest studies on the MAO-inhibitory activities of flavones (chrysin) and isoflavones (genistein). These results revealed that substitutions on the chromone ring at position 3 enhance the MAO-B inhibitory activity. According to the findings for compounds **186** and **187**, a significant SAR (Figure 15), the methoxy group present at position 6 (R^1^) or 7 (R^2^) of the chromone ring was the difference between compounds **186ii** or **187ii** and **186iii** or **187iii**, respectively, in the selective blocking of MAO-B. This indicates that incorporating a methoxy group at position 6, as in compounds **186ii** or **187ii**, caused MAO-B to be removed from the active site.

In contrast, a methoxy substitution at C_7_, as in compounds **186iii** or **187iii**, led to close binding at the active site. Therefore, compounds **185i** and **186i**, which lack a methoxy group, were expected to exhibit moderate MAO-B inhibition. These results are consistent with their previous analysis of the impact of -OCH_3_ replacement on the chromone ring, which showed that diagonal substitutions on the chromone ring increased the MAO-B inhibitory action.

Takao et al. (2021) examined MAO-A and MAO-B inhibitory activities of several 3-styrylchromone derivatives [64]. Most derivatives inhibited MAO-B, except for compound **209**, which had an OH group at position 4 and a methoxy substitution at position 2 in its chemical structure. The chlorine atoms were located at positions 4 and 2 on the phenyl and chromone rings **207**. It efficiently inhibited MAO-B, with an IC_50_ level of 2.2 μM. Derivative **189** showed the highest MAO-B selectivity, with a selectivity index > 3700. Compounds **189** and **207** were found to be mixed-type, reversible MAO-B blockers, suggesting that tight-binding blocking of MAO-B may be part of their mechanism of action.

**Scheme 12:**
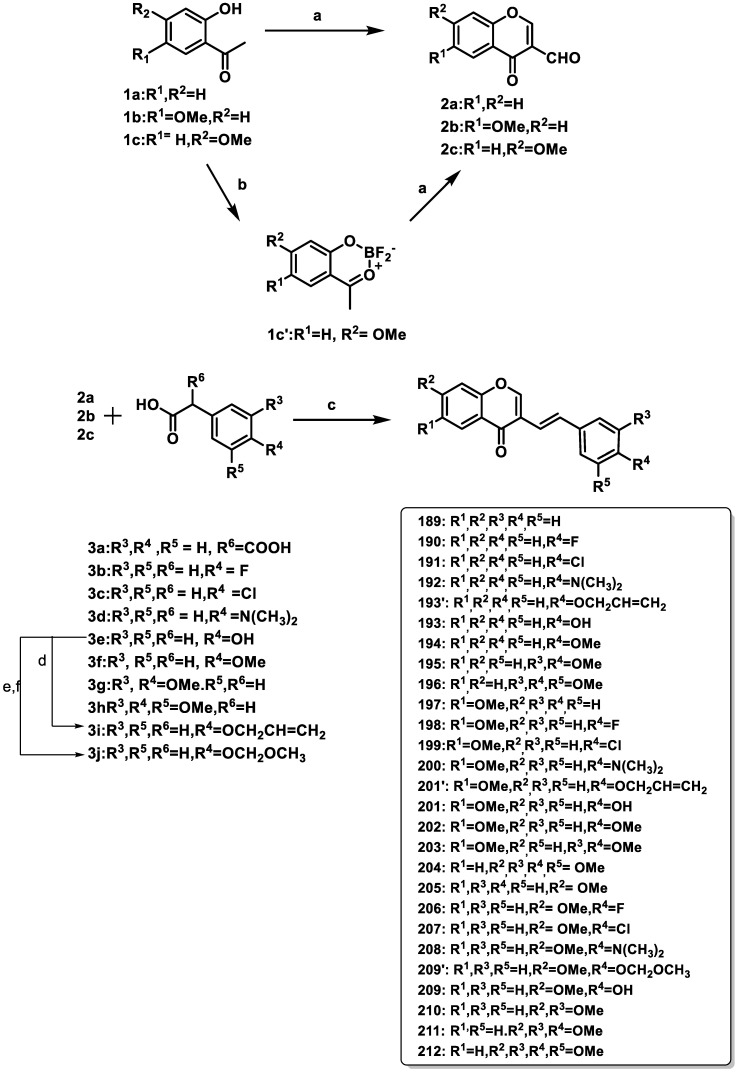
Synthesis of 3-styrylchromone derivatives. Reagents and conditions: (a) DMF/POCl_3_; (b) (CH_3_CO)_2_O/BF_3_·O(C_2_H_5_)_2_; (c) tert-BuOK, dry pyridine, reflux; (d) allylbromide, K_2_CO_3_, EtOH, and then KOH; (e) conc.H_2_SO_4_, MeOH, reflux; (f) chloromethyl methyl ether, K_2_CO_3_, acetone, reflux, and then KOH, MeOH-H_2_O.

As shown in Scheme 12, to create 3-styrylchromone derivatives **190**–**213**, various phenylacetic acid/phenylmalonic acid variants were combined with 3-formylchromone derivatives **IIa**–**c**. The condensation of 3-formylchromone compounds and protected 4-hydroxyphenylacetic acids **IIIi** and **IIIj**, followed by the removal of the protective group, produced 3-styrylchromones with hydroxy groups **193**, **201**, and **209**. Acceptable yields were obtained in all cases. The Vilsmeier–Hack reagent converted 2-hydroxyacetophenone derivatives 1a–c to 3-formylchromone derivatives **IIa**–**c**.

Chromone (4H-1-benzopyran-4-one) derivatives, which are extensively present in natural substances, including 2-styrylchromones, isoflavones, and flavones, are an important category of oxygenated heterocyclic compounds employed in the discovery of drugs. Researchers have developed several novel chromone compounds, such as 2-azolylchromone, 2-styrylchromone, and 3-styrylchromene, and tested their ability to inhibit MAO based on the observations of MAO inhibition by synthetic chromone derivatives. They also provided data on the production and MAO-blocking properties of 2- and 3-cyclicaminochromone analogs. Compared to 2-cyclicaminochromone derivatives, 3-cyclicaminochromone derivatives inhibited MAO-B more effectively. These outcomes led them to explore 3-styrylchromone derivatives comprising recently produced compounds for their MAO-B antagonistic action. This paper describes the synthesis of many 3-styrylchromone variants (Scheme 12) and their inhibitory actions on human MAO-A and MAO-B.

**Figure 16:**
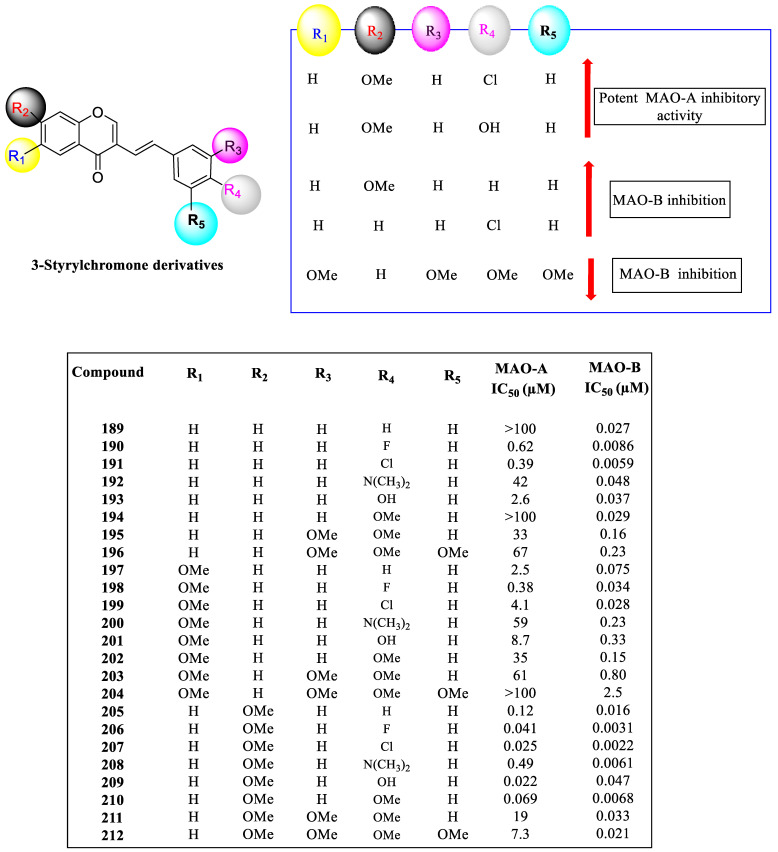
IC_50_ values of 3-styryl chromone derivatives for the inhibition of MAO-A and MAO-B.

The inhibitory actions of MAO-A and MAO-B were tested on all the synthetic 3-styrylchromone derivatives **189**–**212**, as shown in Figure 16. Structure–activity connections were discovered by studying the impact of the substituent on the phenyl ring at positions R_3_, R_4_, and R_5_, the effects on the chromone ring at positions R_1_ and R_2_, and their effects on MAO inhibitory activity.

The 3-styrylchromone derivatives **190**, **191**, **198**, **205**, and **208** had IC_50_ values for MAO-A below 1 μM. In contrast, compounds **206**, **207**, **209**, and **210** had IC_50_ values below 0.1 μM, showing that adding a substituent at position R_2_ as well as position R_4_ was successful in MAO-A inhibition. Derivatives **207** and **209** strongly inhibited MAO-A, with IC_50_ values of **25** and **22**, respectively, compared to the positive control clorgyline, which had an IC_50_ value of 4.9 μM. Except for compound **209**, all tested 3-styrylchromone derivatives had much lower IC_50_ values for MAO-B than for MAO-A. Compounds **206** and **207** showed significantly low IC_50_ values for MAO-B, with 3.1 and 2.2 μM, respectively, when contrasted with safinamide, which was a positive reference. This demonstrates the potential benefit of inhibiting MAO by substituting positions R_2_ and R_4_.

## 4. The Role of 3-Styryl Chromones’ Substituents in Inhibiting MAO-B

MAO-B was significantly inhibited by methoxy substitutions at positions R_1_, R_2_, or R_3_. A comparison was made between the pIC_50_ values of compounds with hydrogen and those of analogs with methoxy substitutions at positions R_1_, R_2_, or R_3_. The results showed that the OCH_3_ groups at positions R_1_ and R_3_ reduced MAO-B inhibition, whereas the methoxy groups at position R_2_ boosted it. The substituent at position R_4_ tends to increase the MAO-B inhibitory activity, which is also true for position R_4_ chloride. Compounds **196**, **204**, and **212** with substituents at position R_5_ appeared to reduce inhibitory activity. According to Takao et al., the phenyl rings of 2-styrylchromone and 3-styrylchromene were strengthened by adding chlorine, which improved their capacity to inhibit MAO-B.

Zhang et al. developed, synthesized, and assessed several chromone-hydroxypyridinone hybrids (Table 6) as potential multimodal anti-AD ligands [65]. Compound **216** exhibited selective hMAO-B inhibitory activity, with an IC_50_ value of 67.02 nM. Derivative **216** considerably improved scopolamine-induced cognitive impairment in AD mice by crossing the BBB.

**Figure 17:**
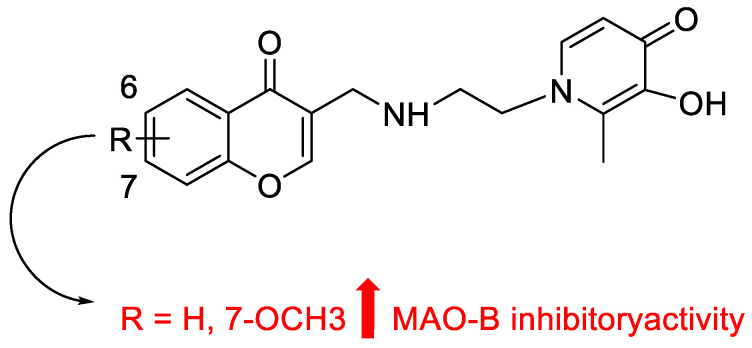
SAR study of chromone derivatives.

The most promising chromone derivatives, with IC_50_ concentrations ranging from 0.067 to 0.088 μM, were modified by alkyl groups at C-6 or C-7 of the chromone nucleus (Figure 17). Compared to the reference medication (pargyline), compound **216** showed the strongest inhibitory action.

## 5. Conclusions

PD therapy was the indication for when MAO-B inhibitors were first made available, and they are still a frequently prescribed cornerstone of therapy. Although not conclusive, the pre- and post-clinical evidence in support of a neuroprotective and disease-modifying effect for MAO-B inhibitors is unmatched by any other class of antiparkinsonian drugs to date. An additional reason to begin using MAO-B inhibitors early in the course of the disease and to keep using them over the long term is the potential that they may reduce the progression of PD. New medicines having MAO-B inhibitory as well as non-dopaminergic activity are still being developed and tested due to the potential of neuroprotection. Considering the limited number of MAO-B blockers currently approved for clinical application, many research projects have focused on developing new MAO-B blockers with greater efficacy. This review focused on the effects of various chromone ring substituents on MAO-B inhibition. The inhibitory effect of MAO-B on chromones has been explored in a small number of studies. Chromone is a MAO-B antagonist. The following is a summary of the SARs for the inhibition of MAO-B by chromone classes:

- The methyl group at the R_4_ position of chromone was shown to be a beneficial substitution.
- The R_4_ position of the chromone with a bromine group demonstrated a large increase in MAO inhibition and fluorine substitution at R_2_ of the phenyl ring.
- NO_2_ and CH_3_ groups in R_3_ decreased MAO activity, whereas the electronegative halogens chlorine and fluorine caused a marked increase in activity.
- Electronegative groups, such as Cl and F substitutions at the para position of styryl chromones, demonstrated higher MAO-B inhibition, as seen in the cases of compounds **207** and **206**.
- The amino chromone derivatives exhibited more powerful MAO-B inhibition than the ester derivatives **126** and **131**. As demonstrated for compounds **131** and **132**, phenyl-to-benzyl chain elongation increased MAO-B inhibition, but further chain elongation diminished activity, as seen for compound **135**. The inhibition of MAO-B was reduced by compounds containing pyridyl side chains.
- Derivatives with meta substituents on the exocyclic ring showed increased potency.
- In the future perspective of view, researchers could modify and extend the alkyl chains at the R_1_ and R_5_ positions of the chromone ring to develop potent MAO-B inhibitors. The introduction of heterocyclic-based amide on the C-3 position of chromone was not explored so far for the development of MAO-B inhibition. This information will be beneficial for discovering and creating a novel category of powerful and specific MAO-B blockers based on chromones.

## Figures and Tables

**Table 1:** MAO inhibitory activities of benzopyrone derivatives **32**–**45** and **52**–**65**.

Compound	IC_50_ (μM) hMAO-B	Compound	IC_50_ (μM) hMAO-B
32	15.32 ± 1.02	52	21.35 ± 1.10
33	7.52 ± 1.05	53	17.10 ± 1.17
34	5.07 ± 1.25	54	4.20 ± 1.08
35	45.40 ± 1.30	55	78.22 ± 1.30
36	13.90 ± 1.30	56	151.6 ± 5.14
37	11.08 ± 1.20	57	45.42 ± 2.32
38	621.70 ± 1.8	58	512.6 ± 2.81
39	5.95 ± 1.28	59	41.8 ± 2.2
40	47.24 ± 1.12	60	21.80 ± 1.21
41	9.03 ± 1.07	61	3.94 ± 1.08
42	228.6 ± 1.26	62	113.5 ± 1.10
43	19.43 ± 1.19	63	210.8 ± 8.1
44	18.90 ± 1.01	64	10.31 ± 1.55
45	*	65	674.2 ± 1.72
Deprenyl	16.73 ± 1.48	Safinamide	23.07 ± 2.07
Rasagiline	49.66 ± 2.26	Clorgyline	*

(*) Inactive at 100 μM.

**Table 2:** Inhibition of MAO by 3-aminomethylidene-2,4-chromandione derivatives.

Code	R	IC_50_ (µM)	
		MAO-A	MAO-B
132	C_6_H_5_	79.6	0.947
133	C_6_H_5_CH_2_	77.9	0.638
134	C_6_H_5_(CH_2_)_2_	101	0.897
135	C_6_H_5_(CH_2_)_3_	312	1.43
136	C_6_H_5_(CH_2_)_4_	155	142
137	_4_Cl-C_6_H_5_	72.1	3.08
138	4-Cl-C_6_H_5_cH_2_	288	NI
139	3-Cl-C_6_H_5_(CH_2_)_2_	NI	NI
140	C_5_H_4_N	73.1	38.7
141	C_5_H_4_N(CH_2_)_2_	41.6	16.66

NI, no inhibition at a maximum tested concentration of 100 μM.

**Table 3:** Inhibition of MAO by 2-styryl chromone derivatives.

Compound	R^1^	R^2^	R^3^	R^4^	TC_50_ (µM) MAO-A	IC_50_ (µM) MAO-B
**142**	H	H	H	H	0.95	0.24
**143**	H	H	H	F	0.59	0.17
**144**	H	H	H	Cl	0.29	0.079
**145**	H	H	H	Br	0.33	0.069
**146**	H	H	H	OMe	2.3	0.049
**147**	H	H	OMe	OMe	25	2.8
**148**	OMe	H	H	H	0.20	0.18
**149**	OMe	H	H	F	0.12	0.064
**150**	OMe	H	H	Cl	26	0.017
**151**	OMe	H	H	Br	0.53	0.024
**152**	OMe	H	H	OMe	0.21	0.19
**153**	OMe	H	OMe	OMe	21	0.68
**154**	H	OMe	H	H	26	0.22
**155**	H	OMe	H	F	35	0.12
**156**	H	OMe	H	Cl	>100	0.27
**157**	H	OMe	H	Br	>100	0.45
**158**	H	OMe	H	OMe	72	1.4
**159**	H	OMe	OMe	OMe	>100	10

**Table 4:** Inhibitory activity of donepezil-chromone hybrids.

		hMAO-A	hMAO-B
Compound	R	IC50 (µM)	IC50 (µM)
**160**	H	63.8	17.68
**161**	6-OCH_3_	7.5	46.27
**162**	6-OBn	11.6	0.035
**163**	6 cH_3_	48.1	19.46
**164**	6-Br	35.5	22.82
**165**	7-OCH_3_	37.4	28.64
**166**	7-Br	31.6	19.73
**167**	H	29.7	33.29
**168**	H	68.6	35.29
**169**	6-OCH_3_	72.4	11.23
**170**	6-OBn	67.2	0.272
**171**	6-CH_3_	30.6	52.45
**172**	6-Br	48.2	26.13
**173**	7-OCH_3_	32.4	30.93
**174**	7-Br	30.3	29.46
**175**	H	29.5	20.03

**Table 5:** Inhibition of MAO by 2- or 3-(N-cyclic amino)chromone derivatives.

Compound	R_1_	R_2_	IC_50_ (µM)	
			MAO-A	MAO-B
2-(N-Cyclic amino)chromone				
**177i**	H	H	38	59
**177ii**	OMe	H	16	27
**177iii**	H	OMe	57	36
**178i**	H	H	31	22
**178ii**	OMe	H	4.1	5.6
**178iii**	H	OMe	8.8	7.4
**183i**	H	H	38	22
**183ii**	OMe	H	2.6	2.8
**183iii**	H	OMe	59	12
**184i**	H	H	66	58
**184ii**	OMe	H	36	40
**184iii**	H	OMe	34	31
3-(N-Cyclic amino)chromone				
**185i**	H	H	23	2.0
**185ii**	OMe	H	23	0.99
**185iii**	H	OMe	18	14
**186i**	H	H	>100	1.5
**186ii**	OMe	H	>100	>100
**186iii**	H	OMe	>100	0.25
**187i**	H	H	>100	0.72
**187ii**	OMe	H	>100	>100
**187iii**	H	OMe	>100	0.015
**188i**	H	H	57	23
**188ii**	OMe	H	34	8.0
**18Siii**	H	OMe	25	7.3

**Table 6:** Inhibitory activity of chromone derivatives against MAO-B.

Compound	R	IC_50_ (μM) hMAO-B
**213**	H	0.0672
**214**	7-methyl	0.0827
**215**	6-methyl	0.0863
**216**	7-methoxy	0.0670
**217**	6-methoxy	0.0886
Pargyline		0.1113

## Data Availability

The datasets used, analyzed, and reviewed were collected from the corresponding authors and online research databases.
